# Estimation of Vehicle Longitudinal Velocity with Artificial Neural Network

**DOI:** 10.3390/s22239516

**Published:** 2022-12-06

**Authors:** Guido Napolitano Dell’Annunziata, Vincenzo Maria Arricale, Flavio Farroni, Andrea Genovese, Nicola Pasquino, Giuseppe Tranquillo

**Affiliations:** 1 Department of Industrial Engineering, University of Naples Federico II, 80125 Naples, Italy; 2 Department of Electrical Engineering and Information Technologies, University of Naples Federico II, 80125 Naples, Italy

**Keywords:** measurements, vehicle dynamics, velocity estimation, machine learning, artificial neural networks

## Abstract

Vehicle dynamics control systems have a fundamental role in smart and autonomous mobility, where one of the most crucial aspects is the vehicle body velocity estimation. In this paper, the problem of a correct evaluation of the vehicle longitudinal velocity for dynamic control applications is approached using a neural networks technique employing a set of measured samples referring to signals usually available on-board, such as longitudinal and lateral acceleration, steering angle, yaw rate and linear wheel speed. Experiments were run on four professional driving circuits with very different characteristics, and the vehicle longitudinal velocity was estimated with different neural network training policies and validated through comparison with the measurements of the one acquired at the vehicle’s center of gravity, provided by an optical Correvit sensor, which serves as the reference (and, therefore, exact) velocity values. The results obtained with the proposed methodology are in good agreement with the reference values in almost all tested conditions, covering both the linear and the nonlinear behavior of the car, proving that artificial neural networks can be efficiently employed onboard, thereby enriching the standard set of control and safety-related electronics.

## 1. Introduction

In recent decades, the development of increasingly advanced automotive safety technologies and collision avoidance assistance systems, the expectation with the intelligent and autonomous transportation system and the growing affluence in individual mobility have been accelerated to enhance not only the performance of active safety technologies against car accidents but also to reduce traffic congestion and improve global traffic efficiency [1,2,3,4]. With end-to-end digitization, beginning with the individual automobile through all elements of the transportation system from road infrastructure to back-end server, vehicles are becoming complex networked mobile computers, including various networked microprocessor-based ECUs that enhance vehicle functionality and provide a general basis for uninterrupted control and management of traffic flow [5,6,7].

ECUs (Electronic Control Units) have increased the level of vehicle dependence on software, and complex control systems have become critical and essential components of the modern development of the vehicle: safety systems, such as the ABS (anti-lock braking system), the TCS (traction control system) and stability control systems, are now available on almost all vehicles [8,9,10,11]. To properly feed the vehicle control systems, the underlying model-based systems must be predictive and reliable; this requires the data acquired from the instrument installed onboard and the enrichment of information provided by the quantities directly measured with sensors through additional signals estimated by means of predictive models to be of high quality [12,13,14,15,16,17,18].

Accurate estimation of vehicle longitudinal speed is one of the most crucial tasks for both the design and implementation of the above safety-related control integrated systems.

Additionally, in the motorsport field, the correct use of tires in terms of grip and wear is a fundamental aspect of improving vehicle performance and ensuring that it can be better than the others. With the aim of understanding in which direction to set the vehicle development and choosing the best vehicle set-up to make proper use of the tires in race situations, it is essential to characterize tires from a dynamic and thermal point of view. That is, it is necessary to determine the forces acting on the tires and their local thermal distribution. Only after having made these characterizations is it possible to reflect on how to maximize the behavior of the vehicle in relation to the tire/road interaction. To carry out these analyses, it is essential to know the longitudinal speed and the sideslip angle of the vehicle. Indeed, reliable knowledge of the actual values of vehicle longitudinal and lateral velocities represents the key factor for an accurate estimation of the tire kinematics, in terms of slip indices [19], i.e., the main control variables to be considered in the most advanced braking and traction control systems [20,21,22].

Unfortunately, direct measurement, also called the “ground reference technique”, of the velocity components requires the adoption of rather complex and quite expensive devices usually not available in ordinary passenger cars; these rely on optical technology, radar sensors, global positioning systems or sensor fusion methodologies. Moreover, such instrumentation may suffer from severe occlusions (i.e., the optical sensor may be covered by the snow), lack of texture (while crossing through a tunnel) or, more generally, an imaging system failure. Therefore, in the face of these problems, it is necessary to find alternative solutions.

In accordance with the research present in the literature, two different methods are mainly used to evaluate longitudinal velocity. The first one is to calculate the vehicle velocity starting from the wheel speed and vehicle acceleration channels. The other one is to use an indirect approach employing model-based methodologies [23,24].

Simple direct procedures, such as the maximum wheel speed methodology [25,26], which evaluates the longitudinal vehicle velocity by using the largest wheel speed measurement when braking, are commonly used. The latter just involves wheel speeds, and there is no need to take into account the road friction; however, it is sensitive to measurement noise and may produce a non-negligible error. Another direct method is based on the adaptive nonlinear filter [25] to estimate the longitudinal velocity using the vehicle acceleration or wheel speed information. It is able to adapt to different road conditions, but the quality of the prediction is not ensured. In [26], another strategy evaluating longitudinal velocity as the integration of the vehicle acceleration calculated via wheel acceleration and speed is proposed. A direct methodology that uses both the wheel speed sensor and the vehicle acceleration sensor is disclosed in [27,28,29] with the aim to improve accuracy. In [27,28], an adaptive nonlinear filter has been chosen, which can reduce the sensitivity to wheel speed measurements but cannot ensure an acceptable accuracy on different roads. In [29], to solve this problem, three Kalman filters are used to estimate the vehicle velocity, and their covariance matrix is adjusted through a fuzzy logic.

The disadvantage of this approach is that it can hardly be used in real time due to the delay of the Kalman Filter. In the automotive field, the research on state estimation through Kalman filtering began in the late 1990s [30], when the first extended Kalman filter (EKF) algorithms, based on single-track models, were proposed. The simplified single-track vehicle models have the advantage of requiring less computational effort and parametrization complexity, whereas the Kalman filtering technique compensates for model approximations thanks to the sensors’ feedback. Usually, a trade-off has to be defined between the increased accuracy, obtainable by a more detailed and well-parametrized model, and the computational capability of the system in which the estimation algorithm has to be employed in real-time.

Other methodologies that use both wheel speed and integration of acceleration are explained in [31,32]. These are structured and precise solutions; however, the fuzzy logic is fairly complex for a large number of maneuvers and needs to be taken into account.

Indirect approaches can be split into a force-based method and a sensor signal fusion-based method. The force-based method [33,34,35] uses a tire model to evaluate the forces exerted by the tire against the road and a vehicle model to estimate the vehicle velocity. The more accurate the vehicle model and the tire model are, the higher the accuracy of this method; however, an accurate vehicle/tire model can produce an excessive computational load. The sensor signal fusion-based method, which focuses on a steering situation, uses wheel speeds, longitudinal acceleration and a yaw rate sensor signal to estimate the vehicle velocity. It uses the integration of longitudinal acceleration to estimate velocity; the prediction’s precision is affected by the bias and error of the accelerometer, which always induces an error accumulation.

This paper presents a novel approach for longitudinal vehicle speed evaluation through the use of neural networks, a paradigm of broader machine learning techniques [36,37,38,39]. Automotive engineers and researchers are certainly familiar with this buzzword, and some have even tried neural networks for their specific applications in models, virtual sensors or controllers. Ford Motor Company is one of the pioneers in automotive NN research and development [40,41,42]. An artificial neural network is a set of simple units, called neurons, that communicate with each other by sending signals through connections and are often organized in different layers according to need. In most cases, an artificial neural network is an adaptive system that changes its structure based on information flowing through the network itself during the training or learning phase [43]. In recent decades, the adoption of neural network methods has undoubtedly become relevant in the automotive field, in particular, in autonomous vehicles as a means to estimate a variable’s value from other variables measured with the standard sensors available onboard without using bulky and expensive measurement instruments. The main advantages of this approach include its low computational cost, which allows for easier implementation of the algorithm within the commercial vehicle ECU, and the accuracy of the results, which makes the estimation of particular interest within complex real-time control environments.

The paper is organized by the following structure: In Section 2, a brief overview of the sensors mounted on the vehicle and in the acquired channels is shown. In Section 3, a method based on feed-forward neural networks is presented with particular reference to the strategies adopted to set the ANN properly. In Section 4, a recurrent neural network is set to try to improve the performance of the methods. Finally, in Section 5, a detailed comparison among the different trained networks is shown, with particular attention to the values of key performance indicators’ (KPIs) to define the best solution for the presented case study.

## 2. Equipped Sensors and Acquired Channels

In order to carry out this research activity, a reference vehicle was chosen, in particular, a high-performance vehicle made available by a partner whose name is withheld for confidentiality reasons. This vehicle was equipped with a sensor platform, able to acquire all of the channels necessary to feed the artificial neural network (ANN). For this vehicle, several pieces of information are available, such as the center of gravity position and moment of inertia values, overall dimensions, suspension and engine characteristics as well as tire mounted and aerodynamic coefficients. All of these parameters are useful for checking the quality of the acquired channels, i.e., evaluating the entity of the load transfer.

### 2.1. Equipped Sensors

Some of the acquired channels used to feed the ANN are directly acquired from the vehicle CAN-bus [44]. The CAN-bus (controller area network) is a standard bus vehicle developed to allow micro-controllers and different sensors to communicate with each other’s applications without the necessity of a host computer; it is a message-based protocol able to transmit to the connected devices four types of messages (data, remote, error, overload); however, it transmits only one at a time, which is representative of actual data transmission. If more than one device transmits at the same time, the highest priority device continues the transmission, whereas the others recede. This protocol is essential because it allows the different sensors installed on the vehicle to communicate. In particular, in the presented research, the following channels are acquired from the CAN-bus: the steering angle and the wheel speeds for the four corners. For the acquisition of the yaw rate and the lateral and longitudinal accelerations, an inertial platform is used. This instrument is made from an inertial sensor block with three accelerometers and three gyros (angular rate sensors). Using the global navigation satellite system (GNSS), the instrument is able to make corrections to all of the measured quantities, guaranteeing accurate evaluations of the accelerations, roll, pitch and yaw. Generally, if it is possible, the inertial platform is mounted near the center of gravity (CoG) position to avoid errors during the signal transport to the CoG itself. Finally, to evaluate the longitudinal velocity, which is used as a reference for artificial neural network training, an optical sensor is mounted on the vehicle. This instrument, taking advantage of the Correvit technology, can directly evaluate the vehicle velocity and its components with a non-contact measurement. All of the described sensors are connected to each other thanks to an external data acquisition system that is able to acquire the different types of signals and phase them properly.

Generally, in motorsport applications, it is not always possible to mount the optical sensor on the vehicle during races or the official test session, which leads to the lack of a big amount of useful information about the maximum performance of the vehicle. For classic passenger cars, instead, these instruments are too expensive and delicate to be mounted on all vehicles intended for sale. In this scenario, the proposed methodology, based on the evaluation of the longitudinal velocity through the use of neural networks, starting from the acquired data for which optical sensors are available, represents a good strategy to widen the volume of the usable data to all the cases in which it is not possible to use a fully instrumented vehicle.

### 2.2. Acquired Channels

Thanks to the sensors mounted on the reference vehicle, described in the previous subsection, it is possible to acquire the following signals:

- steering angle [deg];
- lateral acceleration [m/s^2^];
- longitudinal acceleration [m/s^2^];
- yaw rate [deg/s];
- wheels speed [m/s];
- vehicle longitudinal velocity [m/s].

**Figure 1:**
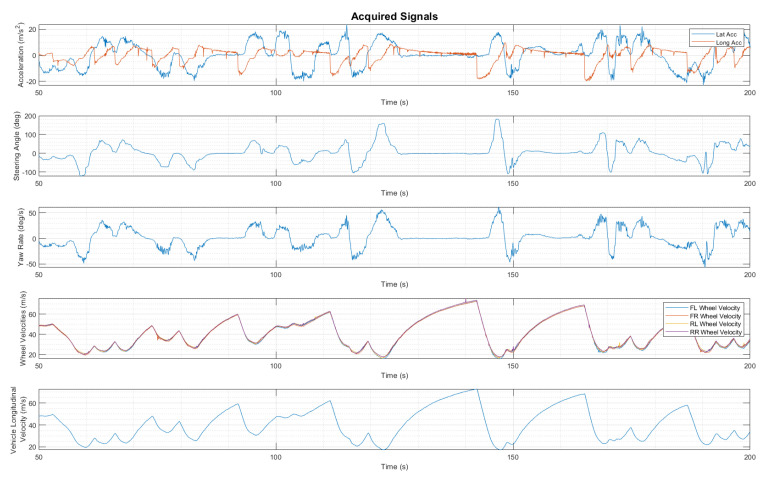
Acquired signals.

These channels are shown in Figure 1.

In order to explore a wide range of operating conditions and different scenarios, the reference vehicle was tested on four different tracks characterized by different road conditions. In addition, to take into account the variable behavior of the tires, these are tested on each circuit in three different conditions: new, averagely worn and worn.

For each acquired run, the final target is to acquire data in the widest range of possible operating conditions. To this aim, long runs are also the best choice to explore the tires’ thermal evolution, starting from the ambient temperature (obtained as a mixed temperature from the air and the track ones), passing through the optimal tire temperature after the warm-up phase, and reaching overheating conditions during the final laps of the run.

Finally, all of the acquired channels have undergone a series of pre-processing operations, such as filtering, offset removals, and changes in units of measurement, in order to check the goodness of the acquisitions and prepare the dataset for the next operation.

## 3. A Feed-Forward Neural Network-Based Longitudinal Velocity Estimation System

Different layouts of a neural network that estimates vehicle longitudinal velocity have been trained, tested and validated against actual measurements obtained from a fully instrumented car on four diverse tracks [45]. Several computational methods for vehicle longitudinal velocity estimation with diverse input dataset configurations and training parameters are presented and compared to the measured signal of interest. In particular, three different configurations of the feed-forward neural networks have been designed and tested [46]: “all wheels”, “front axle wheels” and “rear axle wheels”. The networks have then been trained with different dataset configurations to evaluate the impact on the performance estimation: “all track”, “exclude” and “include”.

In the next section of the paper, the possible improvements to the vehicle longitudinal velocity estimation algorithm employing a recurrent neural network are discussed [47]. The obtained outputs display that the presented methodology may provide good estimation of longitudinal velocity in a large range of possible dynamic maneuvers, covering both linear and nonlinear behaviors of the car and even adopting a limited number of input samples. Furthermore, the authors aim to highlight that an infinite data pool that is useful for training the neural network may even be useless, inasmuch as the results’ accuracy reaches a threshold convergence value after a certain amount of input samples. Hence, there is a need to identify the best match in terms of the methodology performance and costs for planning a test campaign, which could involve a wide range of vehicle physical operating conditions, which employ the commercial applications of artificial neural networks.

Machine learning gathers a subset of tools within broader artificial intelligence-oriented methodologies, whose goal is, in general, to comprehend data structure and fit those data, thanks to models that can be used for different purposes. Although machine learning is part of the computer science field, it is quite different from traditional procedures. Indeed, traditional computing algorithms are a sequence of explicit instructions understandable by computers that are useful for making calculations or finding solutions to several problems. Machine learning procedures, instead, allow computers to train on data inputs and use statistical analyses in order to obtain output values within a specific range [48].

### 3.1. Input Data

The procedures under development are fed by a dataset composed of the following input channels:

- steering angle [deg];
- lateral acceleration [g];
- longitudinal acceleration [g];
- yaw rate [deg/s];
- wheels speed [km/h].

The target variable is:

- vehicle longitudinal velocity [km/h].

The input and output data were acquired from a fully instrumented GT vehicle (undeclared for confidentiality agreements) on four different tracks, as already mentioned in Section 2.

### 3.2. Data Scaling

The input and output variables involved in a data acquisition process are, generally, physical quantities of different types, so they may have really different magnitudes, which could affect the artificial neural network negatively. In fact, when a network is trained on such different data, the convergence and the learning process are slowed significantly, and, in some cases, this may cause the network to non-effectively learn the process [49]. Once the most suitable raw input–output dataset has been selected, it should be pre-processed in order to obtain the correct previsions. Transformation and normalization are two widely used pre-processing methods. Transformation involves manipulating raw data inputs to create a single input to a neural network, while normalization is performed to distribute the data evenly and scale it into a suitable range. Knowing the domain is fundamental to defining pre-processing strategies to highlight underlying features in the data, which can increase the network’s ability to learn the correlations between inputs and outputs. In this paper, the z-score normalization method has been used.

The z-score measures the distance to a data point from the mean in terms of the standard deviation. This is also called data standardization. The standardized dataset is zero mean, has a standard deviation and retains the shape properties of the original dataset (same skewness and kurtosis). For a random variable *X*, the z-score is related to a generic observation $x_{i}$ as $x_{i}$ normalized by the mean μ, and the standard deviation σ is defined as:

(1) $$z_{i}=\frac{x_{i}-\mu }{\sigma }.$$

However, we can usually only obtain the estimate $\bar{x}$ (sample mean) for μ and *s* (positive square root of the sample’s variance) for σ, which are as significant as the sample and are defined as:

(2) $$\bar{x}=\frac{1}{n}\cdot \sum_{i=1}^{n}x_{i}$$

(3) $$s=\sqrt{\frac{1}{n-1}\cdot \sum_{i=1}^{n}{\left(x_{i}-\bar{x}\right)}^{2}}.$$

### 3.3. Preliminary Neural Network Design

To develop a procedure that allows for the evaluation of vehicle longitudinal velocity using machine learning techniques, an artificial feed-forward neural network was designed using the Neuralnet library from RStudio software. A feed-forward neural network is an ANN in which the connections among the units do not create any kind of loop. It is a network in which the information flow is monodirectional from the input layer through the hidden layers (if there are any) and, finally, to the output layer.

In our case, only one hidden layer is used, which is capable of approximating a continuous function on a compact subset of $R^{n}$ under mild assumptions on the activation function. This allows for a non-complex system that can be trained with a low computational burden [50]. To avoid underfitting/overfitting and to achieve a high level of precision, it was chosen, based on the experience of the authors, to have a number of neurons calculated as:

(4) $$N=1.25\;n_{\mathrm{input}}$$

The neurons’ initial weights are not defined because the correlation between the input and output is not preventively known, so they were defined randomly by software.

To avoid overfitting, underfitting and a lack of prediction accuracy, the training parameters were defined opportunely by the authors.

To train the neural networks, the resilient backpropagation algorithm Rprop+ [51] was used which, compared to the traditional backpropagation algorithm [52], allows us to speed up the learning and convergence of the network.

### 3.4. Rprop

The use of $w_{ij}$ denotes the ANN’s weight from neuron “j” to neuron “i”, where “E” is an arbitrary error measure, differentiable with respect to the weights. Concerning the bias parameters, they are considered to be weights obtained from an extra input; the superscripts indicate the learning epoch (iteration). In the Rprop learning algorithm, the direction of each weight update is based on the sign of the partial derivative $\partial E/\partial w_{ij}$. Each weight is updated separately during a single step-size. The main difference with respect to other techniques is that the step-sizes are independent of the absolute value of the partial derivative to speed up the learning and guarantee a better convergence of the network. One iteration of the original Rprop algorithm can be divided into two parts. The first part, for the adjustment of the step-sizes, is basically the same for all algorithms employed in this study. For each weight $w_{ij}$, an individual step-size $\Delta_{ij}$ is adjusted using the following rule:

(5) $$\Delta_{ij}^{\left(t\right)}=\left\{\begin{array}{cc} \eta^{+}\cdot \Delta_{ij}^{\left(t-1\right)} & \mathrm{if}\;{\frac{\partial E}{\partial \omega_{ij}}}^{\left(t-1\right)}\cdot {\frac{\partial E}{\partial \omega_{ij}}}^{\left(t\right)}>0\\ \eta^{-}\cdot \Delta_{ij}^{\left(t-1\right)} & \mathrm{if}\;{\frac{\partial E}{\delta \omega_{ij}}}^{\left(t-1\right)}\cdot {\frac{\partial E}{\partial \omega_{ij}}}^{\left(t\right)}<0\\ \Delta_{ij}^{\left(t-1\right)} & \mathrm{elsewhere} \end{array}\right.$$

where $0<\eta^{-}<1<\eta^{+}$.

If the partial derivative $\partial E/\partial w_{ij}$ has the same sign in consecutive steps, the step-size is increased, whereas if it changes sign, the step-size is decreased (the same principle is also used in other learning methods, e.g., backpropagation). The step-sizes are bounded by the parameters $\Delta_{min}$ and $\Delta_{max}$. In [49], the second part of the algorithm, or the update to the weights, is described for the different Rprop versions (e.g., Rprop+).

### 3.5. Analyzed Approaches

To find the optimal architecture, which ensures a very detailed prediction of vehicle longitudinal velocity, different neural networks were trained using different approaches. These are reported and explained in the following:

- All: Data from all laps and circuits are used to train and test the neural network. The system randomly selects the data to use for the training set while the remaining ones are used in the test set;
- Exclude: The neural network is trained with a set in which all data from a specific circuit or lap (either the first, intermediate or last one) are removed. These data are then used in the test set;
- Include: The neural network is trained with a set containing only data from a specific circuit or lap (either the first, intermediate or last one). These data are then excluded from the testing set.

**Figure 2:**
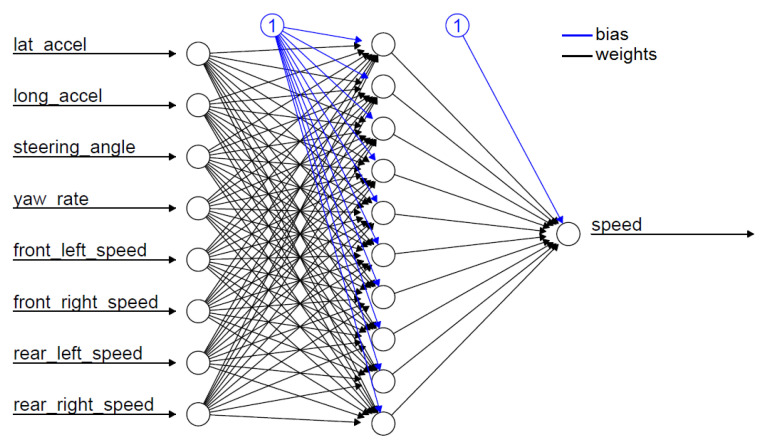
“Four Wheels” Neural Network Configuration.

**Figure 3:**
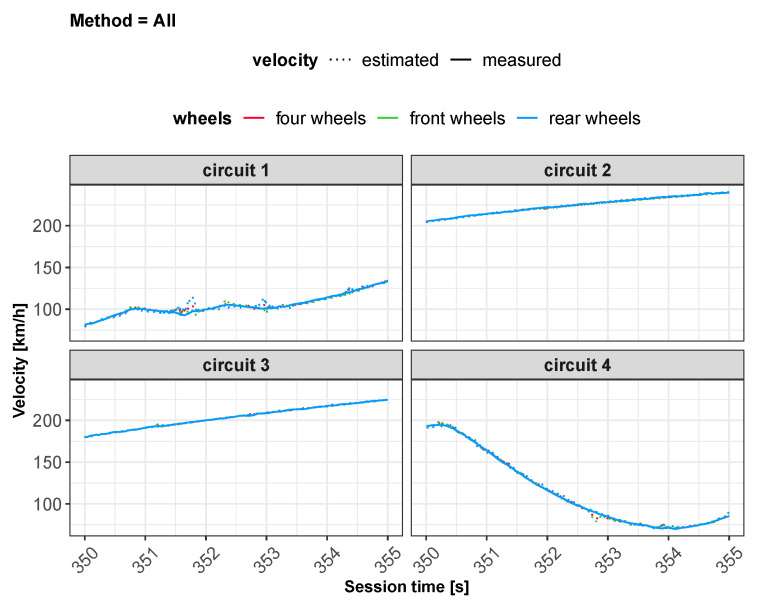
Estimated vs. measured velocity for *All* method.

All of these methods have been applied to three different neural network configurations involving, in order, front wheels, rear wheels and all wheels. The layout of the neural network defined for the “all wheels” configuration is shown in Figure 2. Some preliminary results obtained from the different feed-forward ANNs are displayed in Figure 3 and will be discussed in Section 5.

### 3.6. Models’ Evaluation

To evaluate the performance of the neural network in terms of the estimation error, the classical RMSE was used, while, to compare different configurations of the networks, we used:

1. AIC (Akaike information criterion): The AIC, evaluated on a given dataset, is an indicator of the relative quality of the developed statistical models. Obtained via a series of different models for the data target, the AIC estimates the quality of each model relative to each of the other models. Thus, the AIC provides a mean for model selection [53].
2. BIC (Bayesian information criterion): In statistics, the BIC or Schwarz criterion is a criterion for model selection among a finite set of models which is based, in part, on the likelihood function, and it is closely related to the AIC [54].

## 4. Improvements in Velocity Estimation through a Recurrent Neural Network-Based System

According to what has been reported in the previous section, the “front wheels net” (trained with the “all” method), was used for further investigation, ensuring the highest accuracy compared to the others. In this section, the possible benefits of using a recurrent neural network are analyzed.

### Design of Recurrent Neural Network

**Figure 4:**
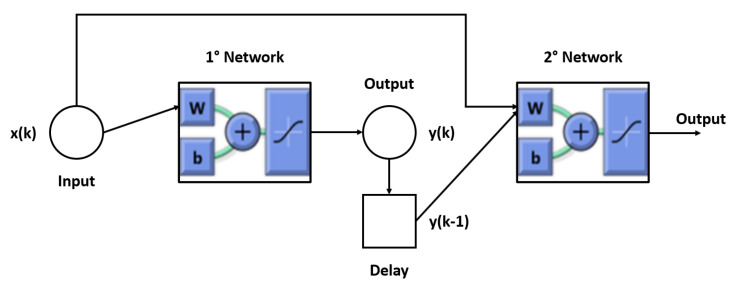
Recurrent neural network layout.

**Figure 5:**
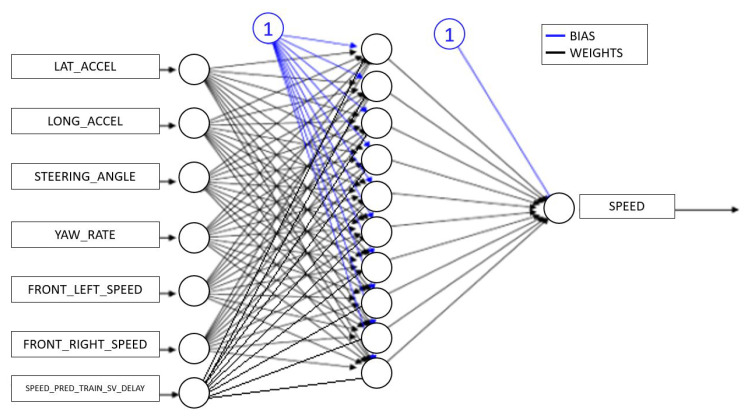
Second net layout and inputs.

Recurrent neural networks (RNN) [47] are a typology of ANNs in which connections among the neurons form a directed graph along a sequence. This allows for the exhibition of dynamic temporal behavior for a time sequence. Different from feed-forward neural networks, RNNs can use their internal state (memory) to process sequences of inputs. In this paper, the RNN was developed as a combination of two different neural networks (Figure 4). The first was trained as explained in the previous section, and the second was trained using the same training parameters but considered the further input to be the longitudinal velocity evaluated by the first net of the previous instance (Figure 5).

## 5. Results

### 5.1. Experimentation Results

Aiming to test and validate the developed procedure, a series of tests were performed whose results will be analyzed and discussed. In the following figures, the results obtained for each method are reported.

**Figure 6:**
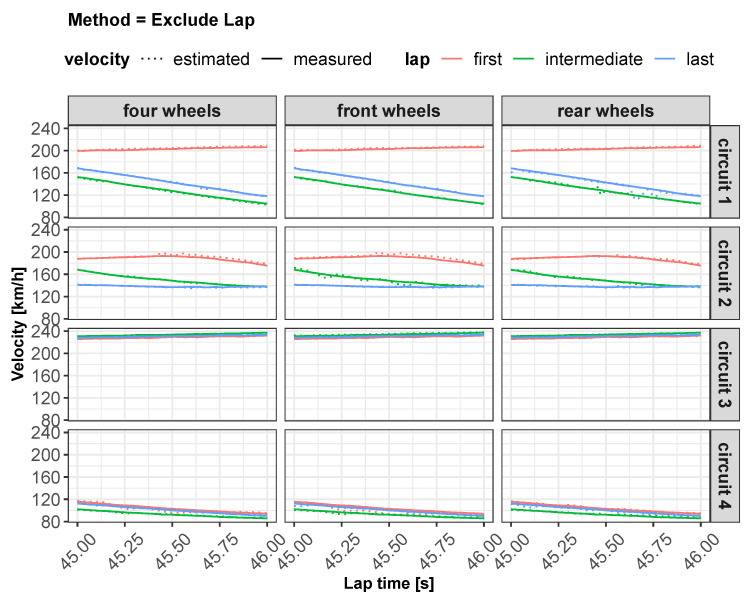
Estimated vs. measured velocity for Exclude Lap method.

**Figure 7:**
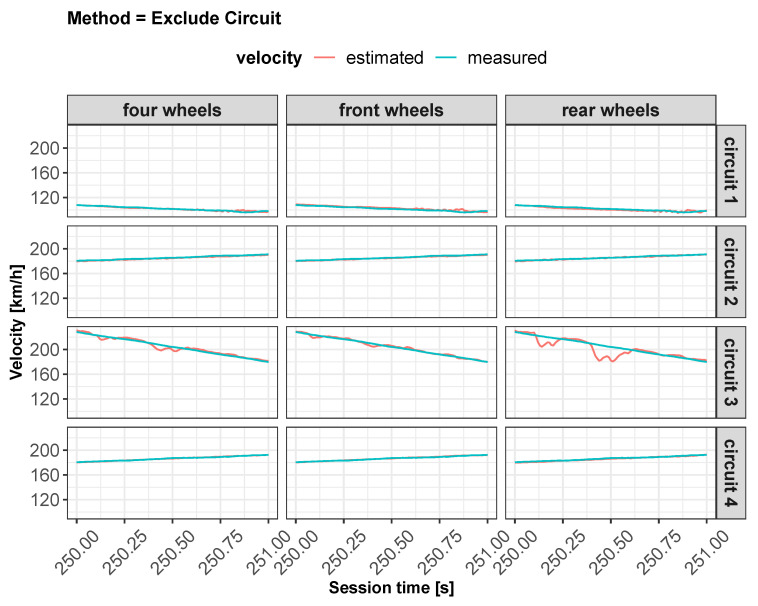
Estimated vs. measured velocity for *exclude circuit* method.

**Figure 8:**
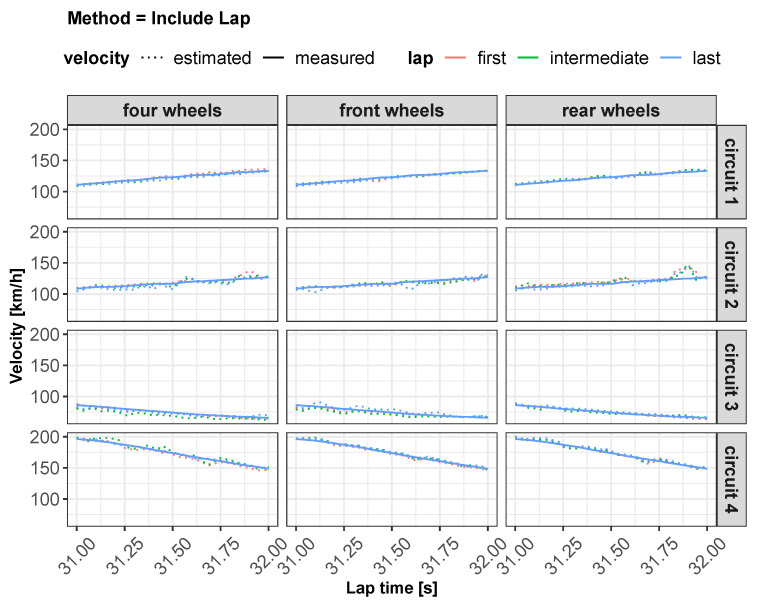
Estimated vs. measured velocity for *include lap* method.

**Figure 9:**
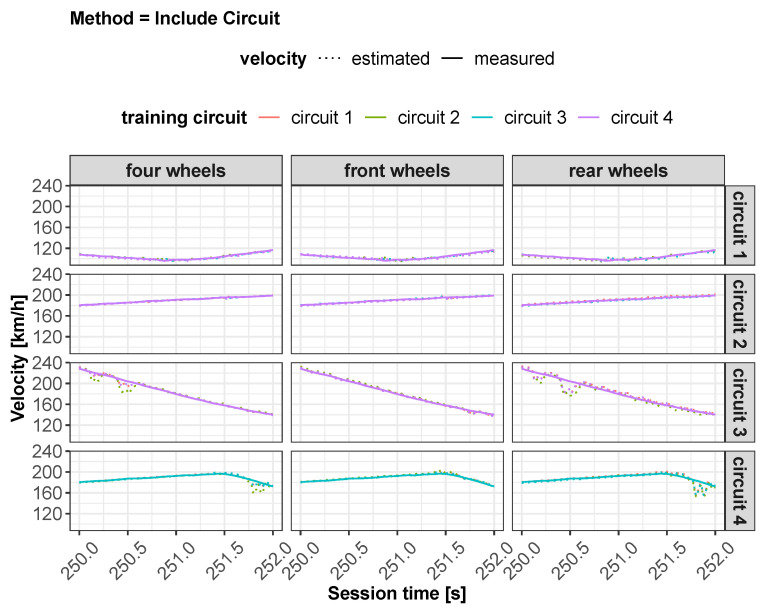
Estimated vs. measured velocity for *include circuit* method.

As concerns vehicle longitudinal velocity, Figure 3 and Figure 6, Figure 7, Figure 8 and Figure 9 report the comparisons between the measured and predicted values, for which the following considerations can be completed:

- The “All” method shows a higher accuracy for each track because it involves a larger set of data, which includes data from all circuits, both for training and the testing phase;
- The “Exclude” circuit method shows a lower accuracy because, in such a case, the neural network is tested on a circuit not used for the testing phase so the different boundary conditions can alter the prediction;
- For each methodology, the highest error is registered for the artificial neural network configuration, which involved the rear wheels (i.e., driving wheels). This is caused by the highest value of slip being reached by the driving wheels, which allows the greatest accuracy. For the following considerations, a neural network that involved no-driving wheels has been considered.

**Figure 10:**
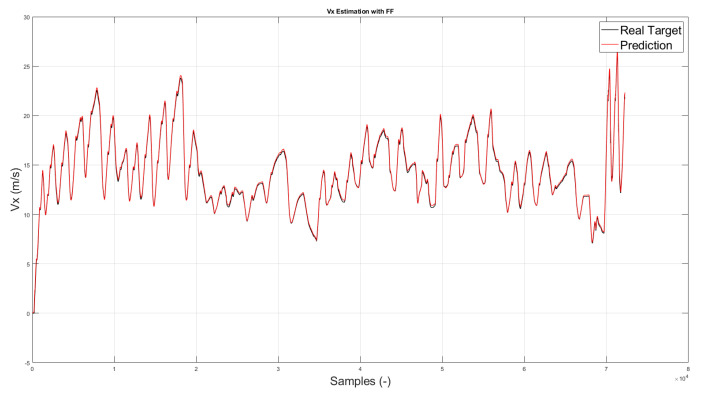
Estimated vs. measured velocity for passenger car.

As explained before, the correct longitudinal velocity estimation is a fundamental goal of vehicle dynamics for different practical applications. For example, in the motorsport field, the knowledge of vehicle longitudinal velocity is essential to understanding the behavior dynamics because, thanks to this quantity, it is possible to estimate the tire slip and, therefore, to combine it with interaction forces [55], to explore and to understand the thermal state of the tire as well; however, in several championship rules, equipping the vehicle with sensors, such as the optical sensor s-motion, to measure such quantity is forbidden, so the estimation is the only way to reach this aim. Moreover, real-time knowledge of the correct vehicle state is needed not only to properly feed the low-level control systems commonly used in commercial cars, such as ABS, ESP and traction control, but also to allow for the development of more accurate advanced driver assistance systems (ADAS) up to fully autonomous driving scenarios. Such control strategies need to have a low computational burden, and the implementation of a neural network may be an essential step. Due to the importance of the estimation of these quantities and to avoid the possibility of obtaining model failure due to different geometrical vehicle designs, inertia and tire properties, the same approach has been tested on a different vehicle in different conditions and on new, fresh and worn tires, to make the authors confident in the proposed methodology. To this aim, in the following figures, the comparison of the velocity acquired by the sensor and the developed NN results is reported for a passenger car (Figure 10).

### 5.2. Feed-Forward ANN KPIs Comparisons

**Figure 11:**
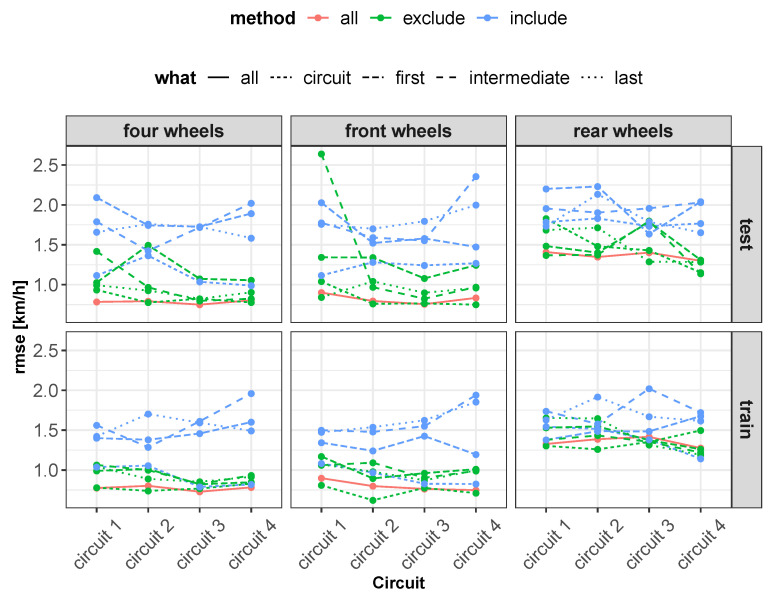
Overall RMSE comparison.

Concerning the results of the RMSE (root mean squared error) (Figure 11), in accordance with what has been reported in the previous plots, it can be observed that the “all” method presents a constant error for all tracks and permits reaching the highest precision of the vehicle longitudinal velocity prediction. This is expected because the neural network has been trained on data coming from each track, so the vehicle behavior is known in all test conditions. These results, in the context of neural networks’ commercial applications, show the importance of identifying a set of tests that allow for the analysis of a wide range of vehicle dynamics conditions.

**Figure 12:**
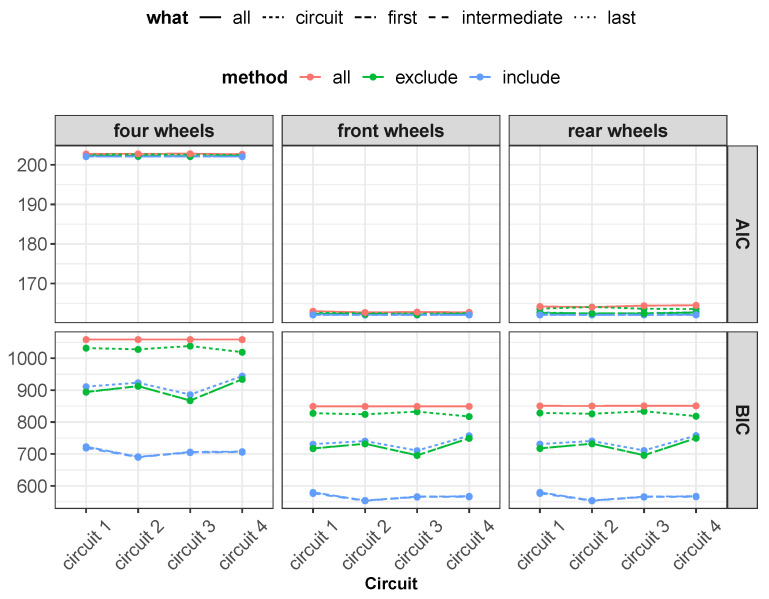
Overall AIC and BIC comparison for all methods.

Subsequently, the proposed models have been analyzed, taking into account their complexity (Figure 12). In terms of model complexity, it is clearly visible that the “four wheels” configuration is the most complex. Comparing the results obtained (Figure 11 and Figure 12), it can be highlighted that the increase in system complexity is not linearly correlated to an increase in system accuracy.

**Figure 13:**
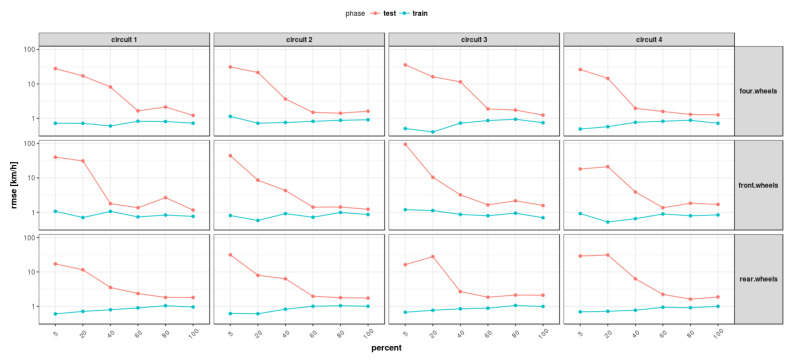
RMSE vs. numerosity of training set.

**Figure 14:**
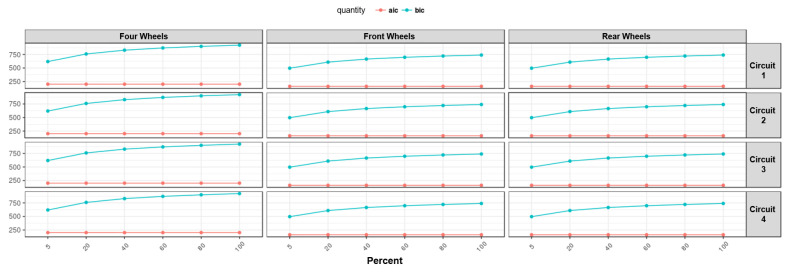
AIC and BIC vs. numerosity of training set.

For all neural network configurations trained with the “All” method, different training conditions were performed with different amounts of data as a percent of the dataset given in the following order: 5%–20%–40%–60%–80%–100% (Figure 13 and Figure 14). In the following figures, it can be observed that the RMSE, BIC and AIC parameters are influenced by the number of samples taken into account in the training phase.

Regarding the number of samples used for the training phase, the aforementioned figures report the comparison between the quality of the neural networks’ performance and the number of samples used for training, for which the following considerations can be completed:

- By increasing the number of training samples after a certain value, the RMSE of the training phase increases as well due to an increase in the constraint condition numbers, which makes the training more difficult.
- Increasing the number of input samples produces an accuracy increment for the testing phase. It is important to notice that after a certain value of training samples, the RMSE of the testing phase reaches a horizontal asymptote.
- The AIC and BIC parameters have higher values for the “four wheels” configuration, which involves more parameters, in accordance with what has been reported previously. By increasing the number of training samples, the AIC and BIC increase as well due to the increment of the constraining condition.
- Concerning the RMSE results, in accordance with what has been reported in the previous analysis, the highest error is registered for the artificial neural networks’ configuration involving the rear wheels (i.e., driving wheels).

Therefore, in the following considerations, the “front wheel” configuration is taken into account, which ensures a lower complexity and a satisfying accuracy in terms of the “MSE”.

### 5.3. Feed-Forward and Recurrent Neural Network Comparison

Different training conditions were performed for both neural networks with different amounts of data as a percent of the given dataset (5%–20%–40%–60%–80%–100%).

**Figure 15:**
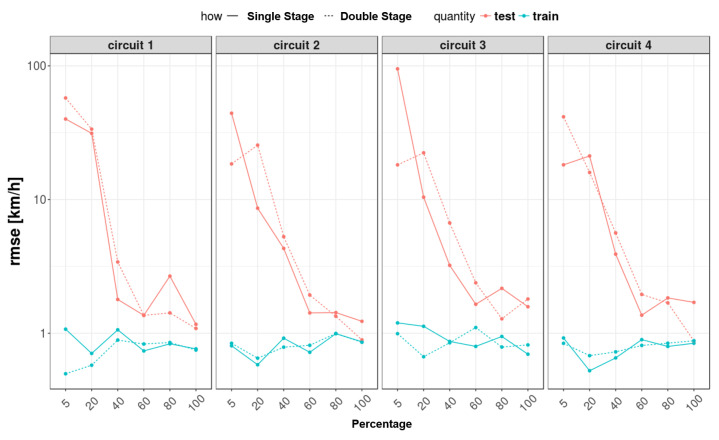
RMSE comparison between feed-forward and recurrent neural networks.

**Figure 16:**
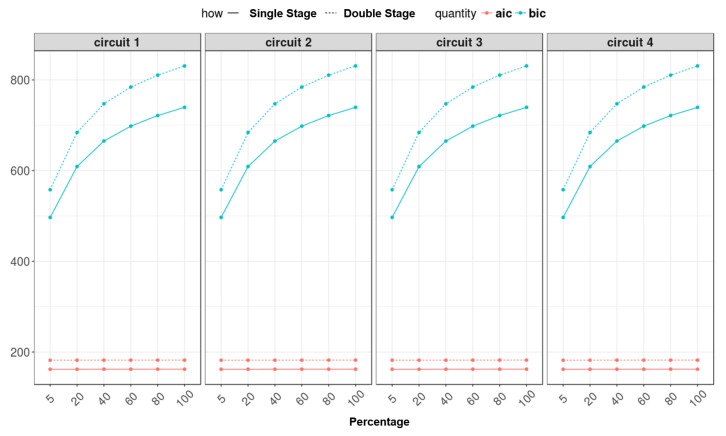
AIC and BIC comparison between feed-forward and recurrent neural networks.

Regarding the RMSE comparison (Figure 15), where, in the plot, the y-axis is on a logarithmic scale between feed-forward and recurrent neural networks, it is observed that there is no noticeable improvement in the results. So, in general, it can be said that the results are similar in both cases and can be considered reliable. This equivalence of results is also due to the fact that the recurrent network was built using a low delay (one sample), which allows for use of the obtained results to feed real-time control systems (i.e., ABS or TCS) and for similarities between the two networks’ topologies. Furthermore, in Figure 16, as predicted, the AIC and BIC parameters are greater in the recurrent neural network, which involves two networks and more parameters.

Given the obtained results, the authors are confident in this approach and with the possibility to use machine learning algorithms to develop virtual estimators of physical quantities (i.e., vehicle longitudinal speed, side slip angle, …) without needing to add the complex and bulky instrumentation with which passenger vehicles are not equipped.

## 6. Conclusions

A vehicle longitudinal velocity estimator based on a neural network has been presented. The outcomes of this work consist of an analytical function in which the inputs are the typical vehicle dynamic signals: steering angle, lateral and longitudinal acceleration, yaw rate and wheel speeds. These signals are easily obtainable from the CAN-bus because all these sensors are commonly mounted on both motorsport and passenger cars. This analysis has been conducted using different approaches. The first one, described in the first part of the paper, regarding the training configurations (“all track”, “exclude” and “include”) and the number of signals used to train the ANN (front wheels, rear wheels and four wheels) in order to find the best set-up able to improve the performance and reduce the model’s complexity. Therefore, three different feed-forward neural networks have been developed and trained using three different training configurations. This analysis shows a minimum RMSE for the “all” method carried out on “four wheels” to train the NN; in particular, the “four wheels”–“all” configuration always shows an RMSE value of less than 1 km/h, whereas, in the other configurations, the RMSE reaches values of more than 2 km/h depending on the tracks considered in the training and validation phases. The second analysis has been carried out in order to understand the best one between the two proposed configurations: the feed-forward or recurrent neural network. The results obtained are really close to those of the classical approach; this is because the feedback signal cannot have a large delay in order to assure real-time working conditions, and the training phase configurations of both the RNN and ANN are the same. In order to obtain a robust methodology, the proposed approach has been tested on both motorsport and passenger cars, on different tracks and with different tire conditions (e.g., new, fresh and worn tires) to exploit the quality of the results because, throughout the entire life cycle, the structural and viscoelastic properties of the tires may change.

The advantages due to the adoption of such procedures are mainly linked to the possibility to provide, by means of the developed analytic algorithms, real-time estimation of a fundamental physical quantity usually measurable only by means of bulky and expensive instrumentation. Moreover, the good agreement between the results obtained by estimation and the acquired vehicle longitudinal velocity, together with the low computational loads required to run the evaluation algorithms, allows us to consider the developed procedures as particularly suitable to be employed in an onboard control logic (e.g., ABS or TCS).

## Data Availability

Not applicable.

## References

[1] Leen G., Heffernan D. (2002). Expanding automotive electronic systems. Computer.

[2] Walta L., Marchau V., Brookhuis K. Stakeholder preferences of advanced driver assistance systems (ADAS)—A literature review. Proceedings of the 13th World Congress and Exhibition on Intelligent Transport Systems and Service.

[3] Tsourveloudis N.C., Valavanis K.P., Hebert T. (2001). Autonomous vehicle navigation utilizing electrostatic potential fields and fuzzy logic. IEEE Trans. Robot. Autom..

[4] Levinson J., Askeland J., Becker J., Dolson J., Held D., Kammel S., Kolter J.Z., Langer D., Pink O., Pratt V. Towards fully autonomous driving: Systems and algorithms. Proceedings of the 2011 IEEE Intelligent Vehicles Symposium (IV).

[5] Charette R.N. (2009). This Car Runs on Code. IEEE Spectr..

[6] Ibañez-Guzmán J., Laugier C., Yoder J.D., Thrun S. (2012). Autonomous Driving: Context and State-of-the-Art. Handbook of Intelligent Vehicles.

[7] Jin Y., Ahn H., Kim K., Choi S., Mueck M., Frascolla V., Haustein T. Adaptive automotive communications solutions of 10 years lifetime enabled by ETSI RRS software reconfiguration technology. Proceedings of the Signal Processing Conference (EUSIPCO), 2017 25th European.

[8] Rajamani R. (2012). Vehicle Dynamics and Control.

[9] Russo R., Terzo M., Timpone F. Software-in-the-loop development and experimental testing of a semi-active magnetorheological coupling for 4WD on demand vehicles. Proceedings of the Mini Conference Vehicle System Dynamics, Identification Anomalies (VSDIA 2008).

[10] Terzo M., Timpone F. (2013). The control of the handling of a front wheel drive vehicle by means of a magnetorheological differential. Int. Rev. Mech. Eng..

[11] Santini S., Albarella N., Arricale V.M., Brancati R., Sakhnevych A. (2021). On-board road friction estimation technique for autonomous driving vehicle-following maneuvers. Appl. Sci..

[12] Lefevre S., Laugier C., Ibanez-Guzman J. Risk assessment at road intersections: Comparing intention and expectation. Proceedings of the 2012 IEEE Intelligent Vehicles Symposium.

[13] Short M., Pont M. Hardware in the loop simulation of embedded automotive control system. Proceedings of the 2005 IEEE Intelligent Transportation Systems Conference.

[14] Zhao P., Chen J., Mei T., Liang H. Dynamic motion planning for autonomous vehicle in unknown environments. Proceedings of the IEEE Intelligent Vehicles Symposium.

[15] Ono E., Hattori Y., Muragishi Y., Koibuchi K. (2006). Vehicle dynamics integrated control for four-wheel-distributed steering and four-wheel-distributed traction/braking systems. Veh. Syst. Dyn..

[16] Halbach S., Sharer P., Pagerit S., Rousseau A.P., Folkerts C. Model Architecture, Methods, and Interfaces for Efficient Math-Based Design and Simulation of Automotive Control Systems. Proceedings of the SAE 2010 World Congress & Exhibition.

[17] Farroni F., Sakhnevych A., Timpone F. (2018). A three-dimensional multibody tire model for research comfort and handling analysis as a structural framework for a multi-physical integrated system. Proc. Inst. Mech. Eng. Part D J. Automob. Eng..

[18] Sakhnevych A., Arricale V.M., Bruschetta M., Censi A., Mion E., Picotti E., Frazzoli E. (2021). Investigation on the model-based control performance in vehicle safety critical scenarios with varying tyre limits. Sensors.

[19] Guiggiani M. (2018). The Science of Vehicle Dynamics.

[20] Romano L., Sakhnevych A., Strano S., Timpone F. (2019). A hybrid tyre model for in-plane dynamics. Veh. Syst. Dyn..

[21] Romano L., Sakhnevych A., Strano S., Timpone F. (2019). A novel brush-model with flexible carcass for transient interactions. Meccanica.

[22] Romano L., Timpone F., Bruzelius F., Jacobson B. (2022). Analytical results in transient brush tyre models: Theory for large camber angles and classic solutions with limited friction. Meccanica.

[23] Villano E., Lenzo B., Sakhnevych A. (2021). Cross-combined UKF for vehicle sideslip angle estimation with a modified Dugoff tire model: Design and experimental results. Meccanica.

[24] Coppola L., De Marco B., Niola V., Sakhnevych A., Timpone F. (2020). Impact attenuator optimum design for a FSAE racing car by numerical and experimental crash analysis. Int. J. Automot. Technol..

[25] Jiang F., Gao Z. An adaptive nonlinear filter approach to the vehicle velocity estimation for ABS. Proceedings of the 2000 IEEE International Conference on Control Applications. Conference Proceedings (Cat. No.00CH37162).

[26] Guofu L. (2004). ABS system is based on data fusion technology, the speed estimation method. J. Sci. Instrum..

[27] Zhao L., Liu Z., Chen H. Sliding mode observer for vehicle velocity estimation with road grade and bank angles adaptation. Proceedings of the 2009 IEEE Intelligent Vehicles Symposium.

[28] Chu L., Shi Y., Zhang Y., Liu H., Xu M. Vehicle lateral and longitudinal velocity estimation based on Adaptive Kalman Filter. Proceedings of the 2010 3rd International Conference on Advanced Computer Theory and Engineering(ICACTE).

[29] Villagra J., d’Andrea Novel B., Fliess M., Mounier H. Estimation of longitudinal and lateral vehicle velocities: An algebraic approach. Proceedings of the 2008 American Control Conference.

[30] Best M., Gordon T., Dixon P. (2000). An Extended Adaptive Kalman Filter for Real-time State Estimation of Vehicle Handling Dynamics. Veh. Syst. Dyn..

[31] Daiss A., Kiencke U. Estimation of vehicle speed fuzzy-estimation in comparison with Kalman-filtering. Proceedings of the International Conference on Control Applications.

[32] Song C.K., Uchanski M., Karl Hedrick J. Vehicle Speed Estimation Using Accelerometer and Wheel Speed Measurements. Proceedings of the International Body Engineering Conference & Exhibition and Automotive & Transportation Technology Congress.

[33] Jaballah B., M’Sirdi N.K., Naamane A., Messaoud H. Estimation of longitudinal and lateral velocity of vehicle. Proceedings of the 2009 17th Mediterranean Conference on Control and Automation.

[34] Hsu L.Y., Chen T.L. (2009). Vehicle Full-State Estimation and Prediction System Using State Observers. IEEE Trans. Veh. Technol..

[35] Farrelly J., Wellstead P. Estimation of vehicle lateral velocity. Proceedings of the 1996 IEEE International Conference on Control Applications held Together with IEEE International Symposium on Intelligent Contro.

[36] Hunt K.J., Irwin G.R., Warwick K. (1995). Neural Network Engineering in Dynamic Control Systems.

[37] Lee J. (1996). Measurement of machine performance degradation using a neural network model. Comput. Ind..

[38] Zhai Y.J., Yu D.L. (2009). Neural network model-based automotive engine air/fuel ratio control and robustness evaluation. Eng. Appl. Artif. Intell..

[39] Meijer G.C. (2008). Smart Sensor Systems.

[40] Jurgen R.K. (2004). Electronic Engine Control Technologies.

[41] Marko K., James J., Dosdall J., Murphy J. Automotive control system diagnostics using neural nets for rapid pattern classification of large data sets. Proceedings of the 1989 International Joint Conference on Neural Networks.

[42] Puskorius G.V., Feldkamp L.A. (1994). Neurocontrol of nonlinear dynamical systems with Kalman filter trained recurrent networks. IEEE Trans. Neural Netw..

[43] Walczak S., Cerpa N. (1999). Heuristic principles for the design of artificial neural networks. Inf. Softw. Technol..

[44] Li Z., Wang D., Kang Q. (2021). The Development of Data Acquisition System of Formula SAE Race Car Based on CAN Bus Communication Interface and Closed-Loop Design of Racing Car. Wirel. Commun. Mob. Comput..

[45] De Martino M., Farroni F., Pasquino N., Sakhnevych A., Timpone F. Real-time estimation of the vehicle sideslip angle through regression based on principal component analysis and neural networks. Proceedings of the 2017 IEEE International Systems Engineering Symposium.

[46] Lippmann R. (1987). An introduction to computing with neural nets. IEEE ASSP Mag..

[47] Yu Y., Si X., Hu C., Zhang J. (2019). A review of recurrent neural networks: LSTM cells and network architectures. Neural Comput..

[48] Goldberg D.E., Holland J.H. (1988). Genetic Algorithms and Machine Learning. Mach. Learn..

[49] Sola J., Sevilla J. (1997). Importance of input data normalization for the application of neural networks to complex industrial problems. IEEE Trans. Nucl. Sci..

[50] Csáji B.C. (2001). Approximation with artificial neural networks. Fac. Sci. Etvs. Lornd. Univ. Hung..

[51] Igel C., Hüsken M. Improving the Rprop learning algorithm. Proceedings of the Second International ymposium Neural Computer, NC’2000.

[52] Connor J.T., Martin R.D., Atlas L.E. (1994). Recurrent Neural Networks and Robust Time Series Prediction. IEEE Trans. Neural Netw..

[53] Portet S. (2020). A primer on model selection using the Akaike Information Criterion. Infect. Dis. Model..

[54] Chen S.S., Gopalakrishnan P. Clustering via the Bayesian information criterion with applications in speech recognition. Proceedings of the 1998 IEEE International Conference Acoustics, Speech and Signal Process, ICASSP ’98 (Cat. No.98CH36181).

[55] Farroni F. (2016). TRICK-Tire/Road Interaction Characterization & Knowledge-A tool for the evaluation of tire and vehicle performances in outdoor test sessions. Mech. Syst. Signal Process..

